# BSR-Seq analysis provides insights into the cold stress response of *Actinidia arguta* F1 populations

**DOI:** 10.1186/s12864-021-07369-9

**Published:** 2021-01-22

**Authors:** Miaomiao Lin, Shihang Sun, Jinbao Fang, Xiujuan Qi, Leiming Sun, Yunpeng Zhong, Yanxiang Sun, Gu Hong, Ran Wang, Yukuo Li

**Affiliations:** 1 Zhengzhou Fruit Research Institute, Chinese Academy of Agricultural Sciences, Zhengzhou, 450000 China; 2 Langfang Normal University, Langfang, 065000 China

**Keywords:** *Actinidia arguta*, Cold resistance, BSR-Seq, Single-molecule real-time sequencing, Cold resistance genes

## Abstract

**Background:**

Freezing injury, which is an important abiotic stress in horticultural crops, influences the growth and development and the production area of kiwifruit (*Actinidia* Lind1). Among *Actinidia* species, *Actinidia arguta* has excellent cold resistance, but knowledge relevant to molecular mechanisms is still limited. Understanding the mechanism underlying cold resistance in kiwifruit is important for breeding cold resistance.

**Results:**

In our study, a population resulting from the cross of *A. arguta* ‘Ruby-3’ × ‘Kuilv’ male was generated for kiwifruit hardiness study, and 20 cold-tolerant and 20 cold-sensitive populations were selected from 492 populations according to their LT50. Then, we performed bulked segregant RNA-seq combined with single-molecule real-time sequencing to identify differentially expressed genes that provide cold hardiness. We found that the content of soluble sucrose and the activity of β-amylase were higher in the cold-tolerant population than in the cold-sensitive population. Upon − 30 °C low-temperature treatment, 126 differentially expressed genes were identify; the expression of 59 genes was up-regulated and that of 67 genes was down-regulated between the tolerant and sensitive pools, respectively. KEGG pathway analysis showed that the DEGs were primarily related to starch and sucrose metabolism, amino sugar and nucleotide sugar metabolism. Ten major key enzyme-encoding genes and two regulatory genes were up-regulated in the tolerant pool, and regulatory genes of the *CBF* pathway were found to be differentially expressed. In particular, a *14–3-3* gene was down-regulated and an *EBF* gene was up-regulated*.* To validate the BSR-Seq results, 24 DEGs were assessed via qRT-PCR, and the results were consistent with those obtained by BSR-Seq.

**Conclusion:**

Our research provides valuable insights into the mechanism related to cold resistance in *Actinidia* and identified potential genes that are important for cold resistance in kiwifruit.

**Supplementary Information:**

The online version contains supplementary material available at 10.1186/s12864-021-07369-9.

## Background

Low temperature drastically influences plant development, productivity and geographic distribution. In recent years, extreme low temperatures have occurred frequently. The kiwifruit industry suffers from an array of threats from low-temperature stress [1]. Therefore, it is important to enhance cold resistance to minimize the economic loss from low temperature injury. Kiwifruit has been domesticated only in the past 100 years, and it has abundant wild resources, which contain excellent cold resistance traits, such as *Actinidia arguta*, which was found to withstand − 38 °C in our previous study [2]. However, the lack of a comprehensive low temperature transcriptome, unexplored cold resistance genes and low temperature signaling hinder our full understanding of cold resistance in kiwifruit. Therefore, identifying cold resistance genes in *A. arguta* is a method for cold resistance breeding and improving kiwifruit cold resistance.

High plants have evolved elaborate mechanisms against cold stress. Cold acclimation is one of the most important mechanisms against low temperature stress in winter. The response of plants to low temperature is a highly complex process involving multiple levels of regulation [3]. A series of physiological and biochemical changes occurred during midwinter in plants [4]. These changes are involved in various pathways and ultimately increase freezing tolerance. The *CBF/DREB1* pathway is a well-studied cold regulatory pathway that plays an important role in cold acclimation in *Arabidopsis*, an adaptive response where plants exhibit increased freeze tolerance after exposure to low nonfreezing temperatures [5–7]. Recent studies revealed that the *CBF*-dependent cold response involves transcriptional, posttranscriptional and posttranslational changes, expanding our knowledge of cold stress regulatory pathways [8]. The role of the starch metabolism pathway in plants under low temperature stress has been widely studied, and sugars accumulate rapidly in plants under low temperature [9]. The source of soluble sugar is generally thought to be from the metabolism of starch in plants [10].

The diploid ‘Hongyang’ was sequenced [11], and the Kiwifruit Genome Database was built [12]; however, *A. arguta* is a tetraploid species, and its polyploid nature and the incompleteness of its genome sequences and annotation limited the transcriptome analysis. Currently, single-molecular sequencing technologies provide an opportunity to thoroughly investigate the molecular mechanisms of the kiwifruit response to low temperature. Single-molecule real-time (SMRT) long-read sequencing technology from Pacific Biosciences (PacBio) is the most popular means of sequencing full-length (FL) cDNA molecules and has been used for whole-transcriptome profiling [13, 14]. FL transcript sequences that eliminate the need for assembly could provide direct information on the transcript isoforms of each gene [15]. SMRT sequencing has also been widely used to predict and validate gene models related to some unique traits. However, the SMRT methodology cannot be directly used to quantify the expression level of transcripts, which may be corrected with next-generation sequencing (NGS) reads [16]. Bulked segregant analysis (BSA) can be used to identify markers linked to any specific gene or genomic region using two bulk DNA pools. Each pool, or bulk, consists of individuals that are identical with respect to a particular trait or genomic region but nonidentical at all unlinked regions [17]. Bulked segregant RNA-seq (BSR-Seq) possesses the advantage of BSA and RNA-seq together, which has the full capability of identifying differentially expressed genes (DEGs) and the ability to identify SNPs between different pools [18]. This method does not require genome information. The BSR-Seq method has been extensively applied to identify major genes in plants such as maize, ginkgoaceae, wheat and cabbage [19–24].

*A. arguta* possesses the strongest cold resistance, which may be involved in its genetic mechanism [25]. However, studies on this species are scarce. Therefore, studies of the genetic mechanism underlying the freezing tolerance trait of this species are still needed. In this study, we used PacBio Sequel and BSR-Seq to identify the DEGs in response to cold stress between tolerant and sensitive pools in *A. arguta* F1 populations. We identified some key genes in starch and sucrose metabolism and regulatory genes related to this pathway. The development of these candidate genes will be the focus of future research, and these results will facilitate the study of the molecular mechanism of freezing tolerance in kiwifruit.

## Results

### Low temperature treatment and evaluation of cold resistance

**Fig. 1:**
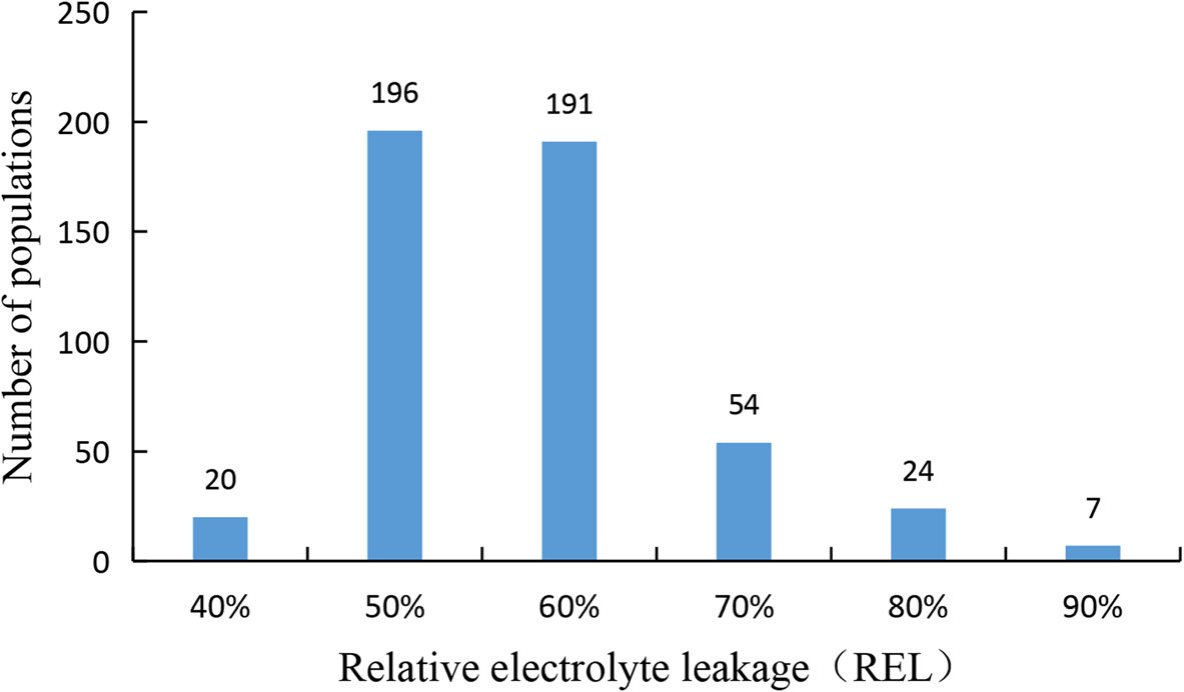
Distribution of REL in populations subjected to −30 °C treatment

Through the cross of ‘Ruby-3’ × ‘Kuilv’ male, a total of 492 populations were obtained, and all the shoots of populations were well planted (Fig. S1). When the dormancy shoots were treated at − 30 °C for 8 h, the REL of populations showed an approximately normal distribution (Fig. 1), and the REL ranged from 42 to 97% (Table S1). Fifty populations with lower RELs (with an average REL of 49%) and 50 populations with higher RELs (with an average REL of 80%) were selected to calculate the LT50. The cold-resistant trait in the populations showed the phenomenon of the superparent. The detailed LT50 is shown in Table 1. Finally, 20 populations with the highest LT50 (tolerant pool) and 20 populations with the lowest LT50 (sensitive pool) were chosen for BSR-Seq analysis. The average LT50 of the 20 higher and lower cold-resistant populations were − 30.52 °C and − 13.97 °C; the highest LT50 was − 36.90 °C, and the lowest LT50 was − 7.51 °C (Table S2).

**Table 1:** LT50 of populations in the tolerant pool and sensitive pool

Tolerant pool			Sensitive pool		
Populations	LT50/°C	Correlation coefficient	Populations	LT50/°C	Correlation coefficient
A-215	−7.51	0.81	B-136	−28.30	0.98
A-169	−10.64	0.86	37	−28.45	0.98
A-216	−12.06	0.72	A-83	−28.50	1.00
A-177	−12.10	0.76	B-123	−28.51	0.98
A-183	−12.35	0.70	B-3	−29.09	0.98
A-120	−12.53	0.84	B-101	−29.11	0.88
A-154	−12.80	0.97	89	−29.18	0.97
A-209	−13.37	0.84	B-52	−29.19	0.84
A-147	−13.39	0.80	85	−29.71	0.98
A-223	−14.40	0.80	B-204	−29.79	0.95
A-240	−14.76	0.94	B-241	−30.05	0.89
A-124	−15.16	0.86	R2–28	−30.90	0.93
B-223	−15.38	0.75	B-161	−30.91	0.92
A-245	−15.58	0.93	A-34	−31.03	0.92
R2–14	−15.62	0.97	B-58	−31.47	0.75
A-191	−15.80	0.93	R2–2	−31.68	1.00
152	−16.14	0.90	13	−32.09	0.98
A-155	−16.22	0.90	A-21	−32.72	0.93
A-247	−16.41	0.82	R2–21	−32.79	0.97
A-168	−17.17	0.94	A-75	−36.90	0.97

### β-Amylase activity and total soluble sugar content

**Fig. 2:**
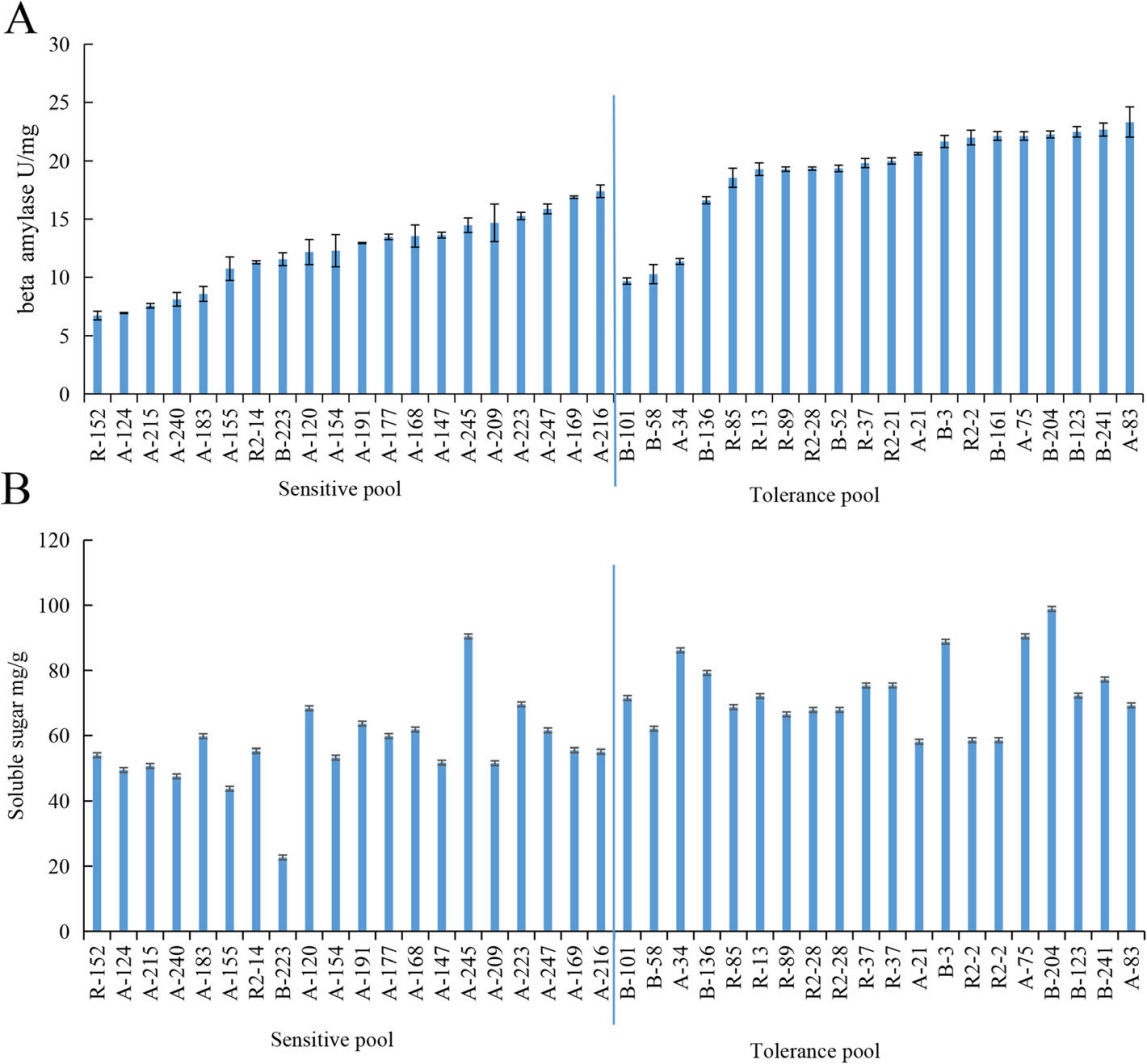
The activity of beta-amylase and the content of soluble sugar in shoots of populations. A: The activity of beta-amylase, B: the content of soluble sugar

β-amylase activity and total soluble sugar content were measured in F1 populations in the tolerant and sensitive pools. The β-amylase activity was higher in the tolerant pool than in the sensitive pool, and the average β-amylase activity in the sensitive resistant and tolerant populations was 12.2 U/mg and 19.15 U/mg, respectively. Soluble sugar showed a higher level in tolerant populations, and the average soluble sugar content in sensitive and tolerant populations was 56.32 mg/g and 75.12 mg/g, respectively (Fig. 2).

### Summary of Illumina HiSeq and PacBio sequel transcriptome sequencing

In total, 328,204,156 raw reads and 315,930,560 clean reads (47.39 G clean bases) with a Q_30_ value of 91.31% were generated in the tolerant pool and 330,407,214 raw reads and 315,880,018 clean reads (47.38 G clean bases) with a Q_30_ value of 91.38% were generated in the sensitive pool by the Illumina HiSeq 2000 platform.

**Fig. 3:**
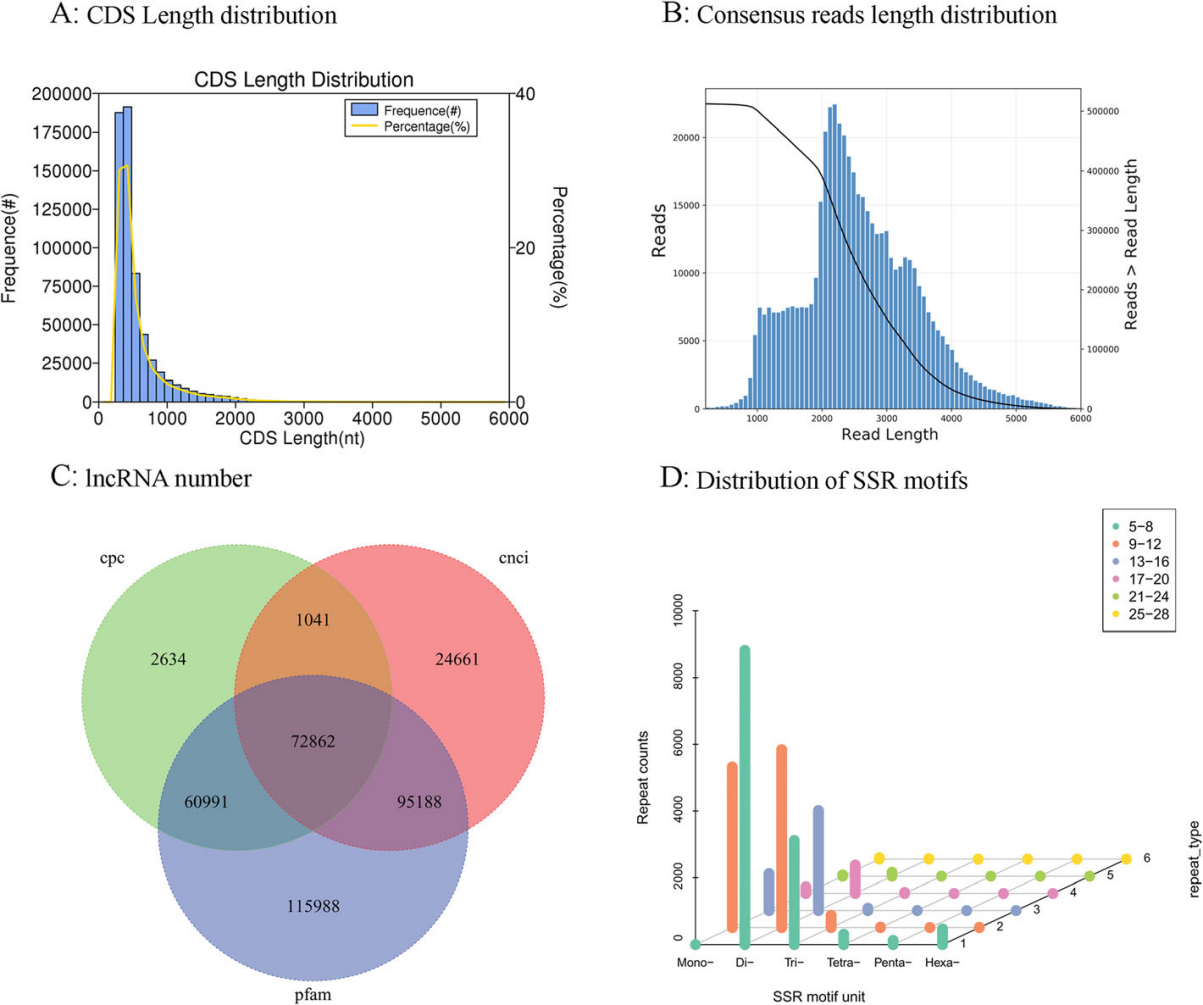
Analysis of the length distribution of CDS and consensus reads, lncRNA number, and distribution of SSR motifs. A: CDS length distribution, B: Consensus read length distribution, C: Venn diagram of lncRNA number predicted by different software packages, cpc is lnc prediction by cpc software, cnci is lnc prediction by cnci sofware, pfam is lnc prediction by pfam sofeware D: distribution of SSR motifs, x axes is the type of SSR, y axes is the number of SSR, z axes is the times of SSR repetition

A total of 1,542,084 circular consensus sequences (CCSs) with a full length of 1,170,272 bp were generated in ‘Kuilv’ male by the PacBio Sequel platform. The full-length nonchimera (FLNC) read number was 1,162,834, with an average length of 2415 bp (Table 2). The PacBio Sequel platform produced a total of 515,285 consensus reads and 13,983,592 subreads (28.33 G bases, with an average length of 2025 bp and an N50 of 2836 bp), which were then corrected using the Illumina reads. The CDS length distributions, consensus read length distributions, lncRNA numbers and simple sequence repeat (SSR) motifs are shown in Fig. 3.

**Table 2:** Summary of the transcriptome data from the PacBio Sequel platform

Item	Number
Number of CCS reads	1,542,084
Average of CCS read length	2551
Full-length reads	1,170,272
FLNC reads	1,162,834
Average FLNC read length	2415
Consensus reads	515,285
Subreads base (G)	28.33
Average subreads length	2025 bp
N50	2836 bp
Total unigenes annotated in at least one database (NR, NT, KOG, Swissprot, Pfam, GO, KEGG)	27,824
Total unigenes	28,496

CCS: circular consensus sequences, FLNC: full-length nonchimera, GO: Gene Ontology, KEGG: Kyoto Encyclopedia of Genes and Genomes, KO: KEGG Ortholog database, KOG: euKaryotic Orthologous Groups, Nr: NCBI nonredundant protein sequences, Nt: NCBI nonredundant nucleotide sequences, Pfam: Protein family

### Functional annotation of unigenes and analysis of DEGs

A total of 28,496 unigenes were obtained for the following analysis. GO classification showed that most unigenes were associated with the metabolic process, cellular process, single-organism process and biological regulation with the molecular functions of binding and catalytic activity. KEGG analysis showed that the top clusters involved unigenes associated with signal transduction, carbohydrate metabolism, folding, sorting and degradation. Annotation against the NR database showed that unigenes in the PacBio transcriptome were identical to *Vitis vinifera* (25.6%), followed by *Sesamum indicum* (7.1%) and *Juglans regia* (7.0%), while the unigenes in the Illumina transcriptome were identical to *Vitis vinifera* (36.6%), followed by *Sesamum indicum* (6.8%) and *Theobroma cacao* (6.0%) (Fig. S2).

**Fig. 4:**
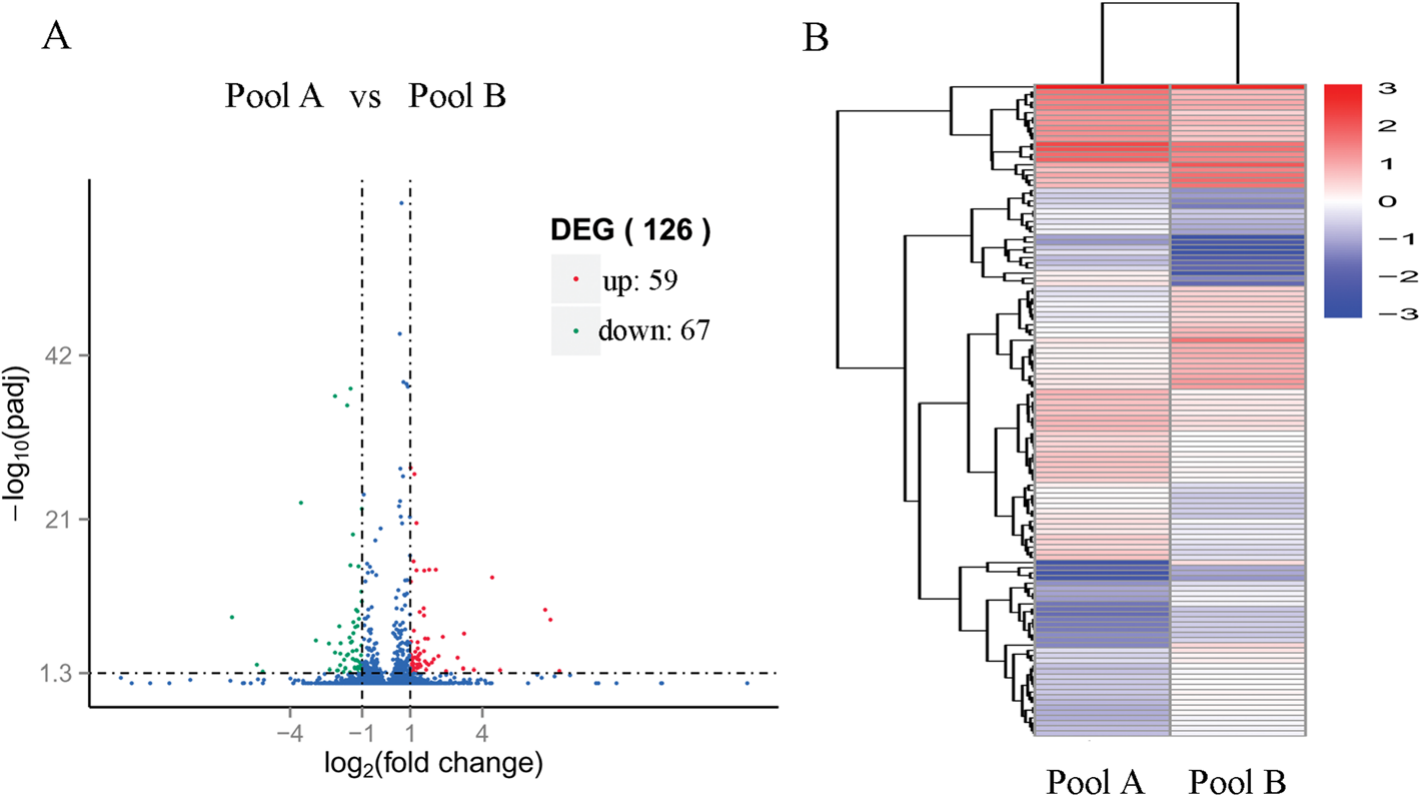
The distribution of differentially expressed genes (DEGs). A: The volcano plot between the sensitive pool (Pool A) and tolerance pool (Pool B); B: Clustering analysis of the DEGs. Blue, down-regulated; red, up-regulated

The DEGs between the tolerant and sensitive pools were also determined. After low temperature treatment, 126 genes displayed significant differential expression between tolerant and sensitive pools, with 59 genes up-regulated and 67 genes down-regulated (Fig. 4).

### GO and KEGG pathway enrichment analysis

**Fig. 5:**
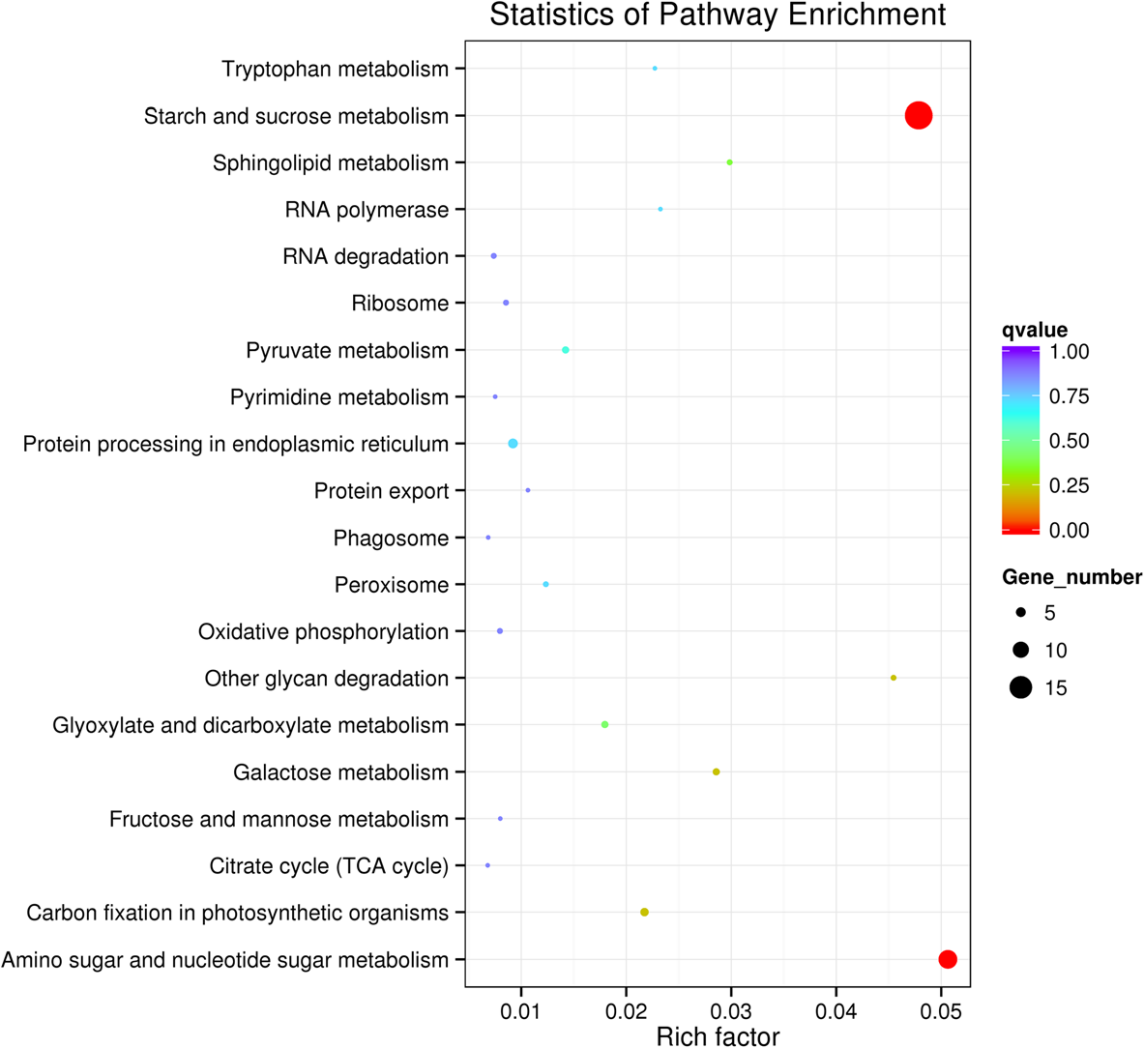
Enrichment of differentially expressed genes in the KEGG pathway

BLAST analysis and GO term annotation were performed to improve our understanding of the functions of these specifically regulated genes. Seventeen GO terms related to biological processes and 5 related to molecular functions (starch synthase activity, transferase activity, transfer of hexosy groups, glucosyltransferase activity and transferase activity) were enriched (Table S3). These DEGs were significantly involved in KEGG pathways, including starch and sucrose metabolism and amino sugar and nucleotide sugar metabolism (Fig. 5). Seven unigenes related to starch and sucrose metabolism were upregulated by low temperature treatment, namely, *AGPase*, *granule-bound starch synthase*, *sucrose synthase* (*SUS*), *1,4-alpha-glucan-branch enzyme* (*GBE*), *alpha-1,4 glucan phosphorylase*, *beta-amylase* (*BAM*), *glucan water dikinase* (*GWD*), and *neutral-alpha-glucosidase and disproportionating enzyme 2* (Table S4).

### Confirmation of differentially expressed genes by qRT-PCR

**Fig. 6:**
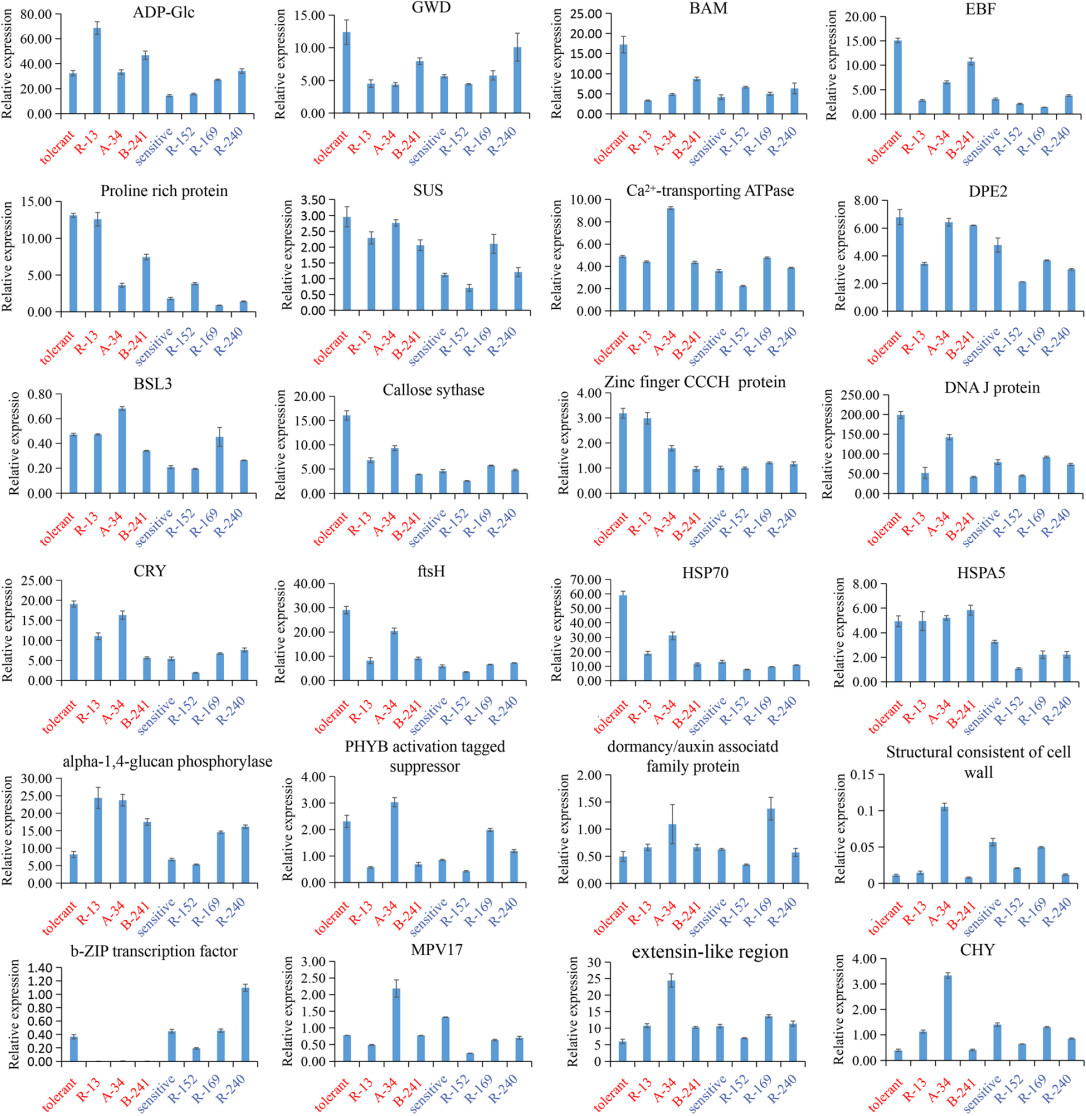
Expression patterns of 9 DEGs between the tolerant pool and sensitive pool and its populations. The results represent the mean ± SE of three replicates

To verify the reliability of the cold responsive gene expression profiles for DEGs, 27 DEGs that contained 18 up-regulated (*ADP-Glc*, *GWD*, *BAM*, *EBF*, *Proline rich protein*, SUS, *Ca*^*2+*^ *transporting ATPase*, *DPE2*, *BSL3*, *Callose sythase*, *Zinc finger CCCH domaint protein*, *DNA J protein*, *CRY*, *ftsH*, *HSP70*, *HPSA5*, *alpha-1,4-glucan phosphorylase*, *PHYB activation tagged suppressor*) and 6 down-regulated genes (*dormancy/auxin associated family protein*, *structrual consistent of cell wall*, *b-ZIP transcription factor*, *MPV17*, *extensin-like region*, *CHY*) were analyzed by quantitative real-time PCR (Table S5). The tolerant pool, sensitive pool and the three randomly selected populations were used as templates. As shown in Fig. 6, the fold change values obtained by qRT-PCR were highly consistent with those based on BSR-Seq data for all of the selected cold responsive genes, despite the difference in the absolute fold change between the two methods. Therefore, some alleles originating from the tolerant pool were preferentially induced to be expressed under low temperature.

## Discussion

*A. arguta* is a deciduous fruit tree, and it is a specie that has a higher cold resistance than other *Actinidia* species. A study on the cold resistance of *A. arguta* was significant for understanding the mechanism of cold resistance in *Actinidia*. Transcriptome analysis has been widely used in studies of kiwifruit, including investigations of fruit development and ripening [26, 27], fruit color [28], and biotic and abiotic stresses, such as waterlogging stress [29] and psa [30]. However, the materials in most studies were cultivars; in this study, F1 populations were used as the materials for the first time. We performed combined transcriptome analysis of NGS and SMRT sequencing and investigated the mechanism in response to low temperature.

*Proline-rich proteins* (*PRPs*) were found to be up-regulated in the tolerant pool of populations, and some evidence suggests that *PRPs* are responsible for cell wall structure, such as *GhHyPRP4*, which may be involved in the plant response to cold stress in cotton [31]; *Brassica BnPRP* genes could be induced by cold [32]; and the *Arabidopsis HyPRP* gene protects the cells during freezing stress [33]. In this study, the higher expression of *PRP* genes, leading to proline accumulation, may be because the increase in proline added mechanical strength to the cell wall and stabilized the structure of organs under low temperature stress [34, 35].

**Fig. 7:**
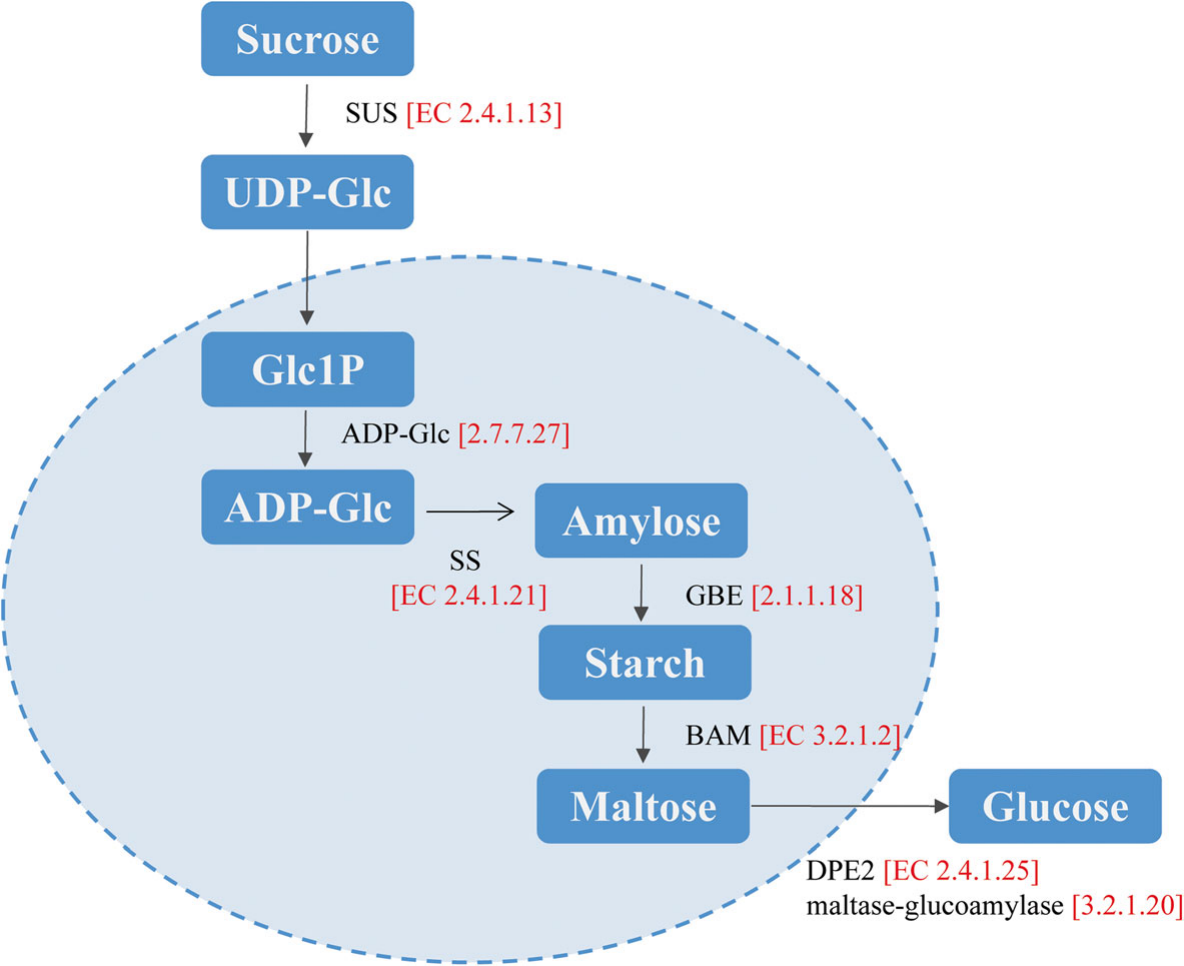
Starch and sugar metabolism in *A. arguta*. All of the genes were up-regulated. SUS, sucrose synthase [EC 2.4.1.13]; ADP-Glc, ADP-glucose [2.7.7.27]; SS, starch synthase [EC 2.4.1.21]; BAM, beta-amylase [EC 3.2.1.2]; DPE2, disproportionating enzyme [EC 2.4.1.25]

The pathways associated with starch and sugar metabolism were significantly enriched. Cold treatment also seemed to trigger enzymes responsible for the production of amylose, starch, maltose, and dextrin [36]. A few key changes in gene expression suggested that these pathways were utilized differently between F_1_ populations with different cold tolerances. Starch is synthesized by *starch synthase* (*SS*) and *1,4-alpha-glucan-branch enzyme* (*GBE*); then, starch is degraded to maltose by *β-amylase* (*BAM*), maltose transfers from plastids to the cytoplasm, and maltose is catalyzed by *disproportionating enzyme* (*DPE2*), whereby one glucose residue is released into the cytoplasm [37]. In our study, *SS*, *GBE*, *BAM* and *DPE2* were all upregulated (Fig. 7). In particular, β-amylase plays an important role in abiotic stress [38]. The β-amylase gene family is induced by low temperature, and *BAM1* and *BAM 3* can degrade starch in *Arabidopsis* [39]. In trifoliate oranges, *PtrBAM1* is induced by low temperatures but repressed by maltose; it is a member of the CBF regulon and plays an important role in cold tolerance by modulating the levels of soluble sugars acting as osmolytes or antioxidants [40]. In our results, the activity of β-amylase was higher in the tolerant pool, and the *bam* gene expression in populations was largely consistent with the activation of the enzyme, suggesting that both the expression and activity of β-amylase are up-regulated by low temperature, which is in agreement with an earlier report [41].

*SUS* was up-regulated in this study. Sucrose transforms the end product D-glucose. Soluble sugars, such as sucrose, glucose, fructose, and raffinose, have been shown to increase in concentration when plant tissues are under cold stress. These sugars are thought to provide freeze protection through stabilization of membranes, scavenging of reactive oxygen species, acting as signaling molecules, and decreasing the freezing point as compatible osmolytes [42]. We detected soluble sugars in F1 populations. The sugar content was higher in the tolerance pool than in the sensitive pool, implying that tolerant populations produced more soluble sugars to resist cold and sugar reserves between nonsoluble and soluble sugar forms when faced with cold temperatures.

In our study, *DnaJ* and *HSP70* were up-regulated in the tolerant pool. The tomato *DnaJ* protein *LeCDJ1* acted as an essential molecular chaperone in protein homeostasis and protein complex stabilization under stress conditions. Heat-shock protein 70 was identified as the partner of *LeCDJ1* [43]. This indicates that *DnaJ* and *HSP70* have essential functions in *A. arguta* against cold temperatures.

**Fig. 8:**
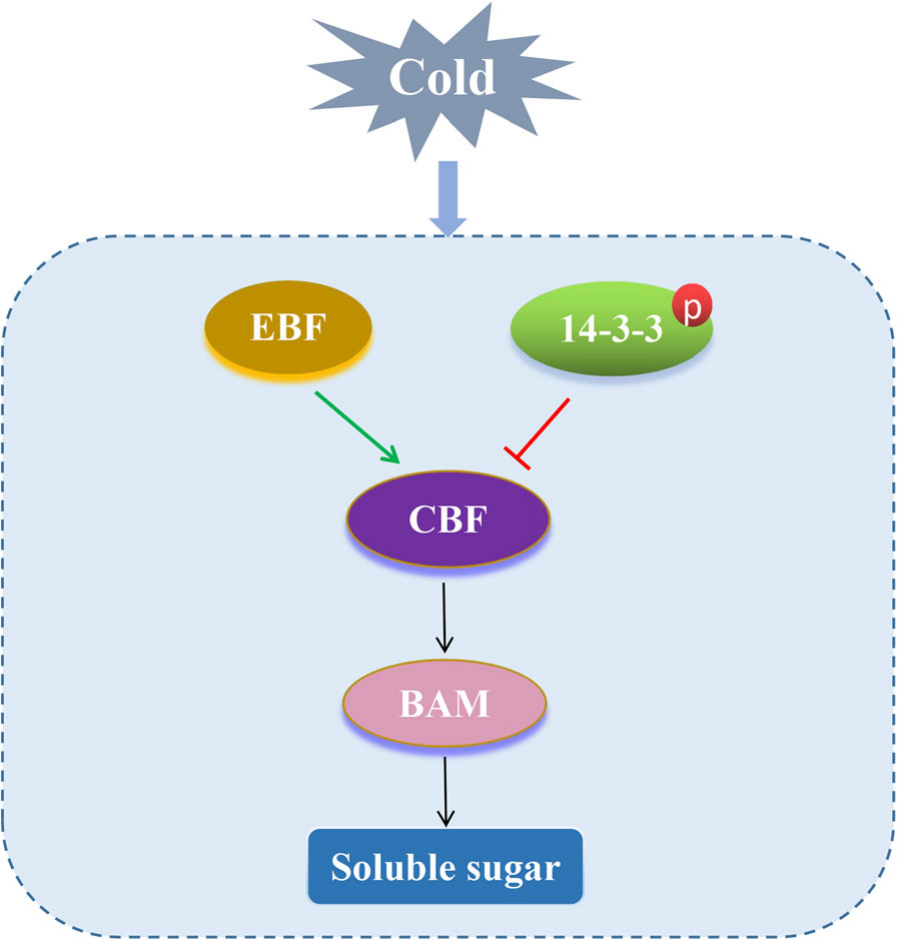
Hypothesized mechanism of cold response in *A. arguta* F1 populations. When kiwifruti encounter cold stress, the cold tolerant populations accumulate more EBF and sensitive populations accumulate more 14–3-3, EBF can achtivate CBF (by increasing CBF transcription) whereas 14–3-3 proteins inhibit CBF by destabilizing CBF through its phosphorylation

Plants have evolved sophisticated strategies in which hormone and cold signaling pathways are coordinated to better adapt to freezing stress. *Ethylene insensitive 3* (*EIN3*) is a key transcription factor involved in ethylene signaling that negatively regulates freezing tolerance in *Arabidopsis*, partially by affecting the expression of *CBFs* by binding to the EBS motifs in their promoters [36, 44]. Two F-box proteins, *EIN3-binding F-box 1/2* (*EBF1/2*), positively regulate *CBF* expression by mediating the degradation of *EIN3* and PIF3 via the 26S proteasome pathway [45, 46]. In this study, *EBF1/2* was upregulated in the tolerance pool, implying that *A. arguta* could increase cold resistance by increasing the expression of *EBF* to regulate the *CBF* gene. Cold perception seemed to differ between the sensitive and tolerance pools. *14–3-3* was up-regulated in the sensitive pool. *14–3-3* family proteins are extensively involved in plant freezing tolerance. Under cold stress, CRPK1 phosphorylates *14–3-3* proteins and triggers their translocation from the cytoplasm to the nucleus; *14–3-3* proteins interact with and destabilize *CBF1/3* proteins, thus attenuating the *CBF* pathway and preventing an excessive cold response [47]. Another study showed that *Arabidopsis* RCI1A, a 14–3-3 protein, negatively regulates freezing tolerance [19]. In our study, in the sensitive pool, the higher expression of *14–3-3* and the phenotype of sensitive cold resistance led us to speculate that *14–3-3* negatively regulates freezing tolerance in *A. arguta*, possibly by destabilizing the *CBF* pathway to stop COR genes (Fig. 8). In addition, we found that the transcription factor *CHY* was up-regulated in the sensitive pool, *CHY* could respond to low temperature in pineapples, and *A. arguta* may try to resist cold in other pathways when the *CBF* pathway is suppressed.

Although some regulators of *CBF* were detected, we also failed to detect *CBF* transcription factors, which is consistent with the results found for *Poncirus trifoliata* [48]. First, most of the genes coding for the regulatory proteins, such as the *CBFs*, exhibited a transient and temporary change under abiotic stresses. At our low temperature (with 8 h), the mRNA abundance of the regulatory genes may be indistinguishable. Second, woody plants differ from herbal plants (such as *Arabidopsis*) when faced with low temperature stress due to their natural variation in the length of the growth cycle [49]. Third, there were nonsignificant differences in the tolerance and sensitive pools under a long-term, low-temperature treatment. The identification of DEGs may be influenced by several factors, such as stress intensity, duration of stress imposition, experimentation strategy and plant species/genotypes [50], and our results also support these conclusions.

## Conclusions

In summary, the biparental F1 populations were constructed. Then, we performed BSR-seq combined with SMRT sequencing in F1 populations. DEGs induced by − 30 °C low-temperature treatment were identified. Low-temperature treatment increased the content of total soluble sugar and the activity of β-amylase in the cold-tolerant pool. Ten key enzyme-encoding genes and 2 regulatory genes were up-regulated. The encoding genes included the following: *AGPase*, *granule-bound starch synthase*, sucrose synthase (*SUS*), *1,4-alpha-glucan-branch enzyme* (*GBE*), *alpha-1,4 glucan phosphorylase*, *beta-amylase* (*BAM*), *glucan water dikinase* (*GWD*), *neutral-alpha-glucosidase, disproportionating enzyme 2* and *proline rich protein* (*PRP*)*.* Regulatory genes included *EIN-bingding F box* (*EBF*) and *14–3-3*. Most of the DEGs were enriched in starch and sugar metabolism and their regulatory pathway. We concluded that starch degradation and soluble sugar synthesis are important for cold resistance in kiwifruit.

## Methods

### Plant materials

F1 populations were generated in 2015 with the cross of ‘Ruby-3’(♀) × ‘Kuilv’ male(♂), and the cross was finished at Zhengzhou Fruit Research Institute. Two parents, ‘Ruby-3’ and ‘Kuilv’ male (2n = 4x = 116), are *A. arguta*; ‘Ruby-3’ originates from Henan Province, and ‘Kuilv’ male originates from Jilin Province. All the populations and parents were planted at the Zhengzhou Fruit Research Institute, Chinese Academy of Agricultural Sciences, China.

### Low temperature treatment

All the populations were planted with well-watered and non-pest plants. Plant materials were the dormancy shoots of F1 populations. Dormancy shoots were treated at − 30 °C for 8 h [2, 51], and relative electrolyte leakage (REL) was calculated to screen for extreme cold resistance. Fifty populations with the highest REL and 50 populations with the lowest REL were selected to evaluate the detailed cold resistance and treated at − 15 °C, − 20 °C, − 25 °C, − 30 °C, − 35 °C for 8 h. Then, the LT50 was calculated. Twenty populations with the higher REL (tolerance pool) and twenty populations with the lower REL (sensitive pool) were chosen, and the shoots were treated with − 30 °C for 8 h and then used for measurement of β-amylase activity, total Soluble Sugar content and BSR-Seq.

### Measurement of electrolyte leakage

After low-temperature treatment, the shoots without buds were cut into 1–2-mm-thick slices. Then, 0.2 g of the slices was incubated in 30 ml of double-distilled water for 2 h with shaking at 200 rpm at room temperature. The initial electrical conductivity (C1) was measured using a digital conductivity meter (DDS-307, Rex, China). The samples were heated to boiling for 30 min and then cooled down to room temperature with shaking for 30 min. The second electrical conductivity (C2) measurement was obtained. The REL was calculated as indicated by Eq. 1:

1 $$ REL\left(\%\right)=\left({C}_1/{C}_2\right)\ast 100\% $$

The FT was expressed as LT50 (half the lethal temperature at which REL reaches 50%) by fitting the response curve obtained by the REL with a logistic sigmoid function (Eq. 2):

2 $$ y=k/\left( 1+{ae}^{\hbox{-} bx}\right) $$

where x is the treatment temperature, y is the REL value, k indicates the extreme value when x approaches infinity, and a and b are the equation parameters. If the correlation coefficient r is close to 1, the equation is used to calculate LT50 [2].

### Measurement of β-amylase activity and Total soluble sugar content

After the 20 tolerant populations and 20 sensitive populations were treated with − 30 °C, β-amylase activity and total soluble sugar content were measurement. β-amylase activity and total soluble sugar content were determined using the relevant kits (Nanjing Jiancheng Bioengineering Institute) according to the manufacturer’s instructions. Each treatment was repeated at least three times with consistent results. Data are presented as the means of three biological replicates ±SE from one representative experiment. The data were analyzed by Duncan’s multiple range tests in the ANOVA program of SPSS (IBM SPSS 22), using *P* < 0.05 and *P* < 0.01 to indicate statistical significance.

### RNA extraction

Forty populations and ‘Kuilv’ male RNA in the dormancy shoots were extracted using an RNA Isolation Kit (HUAYUEYANG, China). The concentration and quality of the extracted RNAs were assessed using the NanoDrop spectrophotometer. Finally, ‘Kuilv’ male RNA was subjected to Pacific Bioscience (PacBio) single-molecule long-read sequencing, while 40 populations of RNA were submitted for second-generation transcriptome sequencing in a flow cell on the Illumina HiSeq platform for BSR-Seq.

### Library preparation and PacBio sequencing

FL cDNA synthesis was completed using the SMARTerTM PCR cDNA Synthesis Kit. Then, PCR amplification, quality control and purification were performed. Size selection was performed using the BluePippin Size Selection protocol. Then, the product was separated into cDNA fractions with lengths of 1–2, 2–3 and 3–6 kb. The cDNA products were submitted for production of SMRTbell Template libraries using the SMRTbell Template Prep Kit. Finally, the three libraries were sequenced using thirteen SMRT cells.

### RNA-seq and bulked Segregant analysis

Tolerance and sensitive pools were generated after − 30 °C low temperature treatment for BSR-Seq analysis. Two extreme pools were constructed using equal amounts of tissues from the same location on the plants at the same growing stage. Shoots collected from the most cold-tolerant individuals and the most cold-sensitive individuals were mixed together and grouped into tolerant and sensitive pools, respectively. Transcriptome sequencing was performed on the Illumina HiSeq 2000 platform (China Novogene, Beijing). The Illumina library was prepared according to the protocol described in the next-generation transcriptome article [52].

### Gene functional annotation and DEG analysis

Seven databases (NR, Swiss-Prot, GO, NT, KOG, Pfam, KEGG) were selected to map the nonredundant transcript sequences and obtain the annotation information of the transcript with e-values of 1e^− 5^ against a total of seven databases [53–58].

RNA sequencing reads were aligned to the abovementioned SMRT transcripts obtained by Trinity [59], and expression levels were estimated using RSEM [60]. The expression levels of the unigenes were expressed as fragments per kilobase of transcript per million mapped read (FPKM) values to eliminate the influences of gene length and sequencing quality difference on the estimated gene expression [61].

Differential gene expression analysis was carried out between the tolerance and sensitive pools using DEGSeq [62]. For the samples without biological repetitions, TMM was used to standardize the readcount data [63], and DEGSeq was then used for differential gene expression analysis. In this study, genes with adjusted *q* values less than 0.005 and fold changes greater than or equal to 1 were identified as DEGs. Gene ontology (GO) term annotation was conducted for functional classification of DEGs [64], and further metabolic pathway enrichment analysis was carried out using KOBAS (2.0) against the KEGG (Kyoto Encyclopedia of Genes and Genomes) database [65, 66].

### qRT-PCR analysis

Shoot tissues of F1 populations were analyzed in three biological replicates. Samples were ground as described in the materials. RNA samples were converted to single-stranded cDNA using the cDNA Synthesis Kit (TOYOBO). Candidate genes were selected from previous data. SYBR Green-based RT-qPCR analyses were performed in a LightCycler 480 (Roche480) on a 96-well plate. The conditions for the PCR amplifications were as follows: 95 °C for 5 min, followed by 45 cycles of 10 s at 95 °C, 20 s at 60 °C, and 20 s at 72 °C. At the end of each experiment, a melt-curve analysis was carried out using the default parameters (5 s, 95 °C and 1 min, 65 °C). *β-actin* in the kiwifruit was considered the control gene for normalization. All analyses were repeated three times using biological replicates. The relative expression levels were calculated using the 2^-ΔCt^ method. RT-qPCR primers of target genes were designed using Primer Premier 5 software (Table S1).

## Supplementary Information

**Additional file 1: Table S1.** Relative electrolyte leakage (REL) of 492 populations subjected to − 30 °C treatment.

**Additional file 2: Table S2.** Lethal temperature of 50% (LT50) in 100 populations of ‘Ruby-3’ × ‘Kuilv’ male.

**Additional file 3: Table S3.** Significant KEGG pathways associated with differentially expressed genes.

**Additional file 4: Table S4.** Significant GO classifications associated with differentially expressed genes.

**Additional file 5: Table S5.** Primers used for qRT-PCR.

**Additional file 6: Fig. S1.** The hybrid of ‘Ruby-3’ × ‘Kuilv’ male.

**Additional file 7: Fig. S2.** Gene functional annotation of unigenes based on PacBio transcriptome data corrected by HiSeq 2000. A: GO classification, B: NR classification, C: KOG function classification, D: KEGG classification.

## Data Availability

The raw reads of the BSR-Seq data in this study have been deposited in the NCBI SRA database under accession number SRR12668267, SRR12668268.

## Abbreviations

BSA: Bulked segregant analysis
BSR-Seq: Bulked segregant RNA-seq
CCSs: Circular consensus sequences
DEG: Differentially expressed unigenes
FL: Full-length
FLNC: Full length non-chimeric reads
GO: Gene ontology
IRs: Inverted repeats
KEGG: Kyoto Encyclopedia of Genes and Genomes
KO: KEGG Ortholog database
KOG: euKaryotic Orthologous Groups
LT50: Lethal temperature of 50%
NCBI: National Center for Biotechnology Information
NGS: Next-generation sequencing
Nr: NCBI nonredundant protein sequences
Nt: NCBI nonredundant nucleotide sequences
PacBio: Pacific Biosciences
Pfam: Protein family
REL: Relative electrolyte leakage
SSR: Simple sequence repeat
SMRT: Single-molecule real-time

## References

[1] Lin M, Sun S, Qi X, Wang R, Fang J (2020). Advances in research on cold resistance in kiwifruit. Journal of fruit science.

[2] Sun S, Qi X, Wang R, Lin M, Fang J (2020). Evaluation of freezing tolerance in Actinidia germplasm based on relative electrolyte leakage. Hortic Environ Biotechnol.

[3] Zhang LL, Zhao TT, Sun XM, Wang Y, Du C, Zhu ZF, Gichuki DK, Wang QF, Li SH, Xin HP (2019). Overexpression of VaWRKY12, a transcription factor from Vitis amurensis with increased nuclear localization under low temperature, enhances cold tolerance of plants. Plant Mol Biol.

[4] Khan TA, Yusuf M, Ahmad A, Bashir Z, Saeed T, Fariduddin Q, Hayat S, Mock HP, Wu T (2019). Proteomic and physiological assessment of stress sensitive and tolerant variety of tomato treated with brassinosteroids and hydrogen peroxide under low-temperature stress. Food Chem.

[5] Wisniewski M, Nassuth A, Arora R (2018). Cold hardiness in trees: a mini-review. Front Plant Sci.

[6] Jaglo-Ottosen KR, Gilmour SJ, Zarka DG, Schabenberger O, Thomashow MF (1998). Arabidopsis CBF1 overexpression induces COR genes and enhances freezing tolerance. Science.

[7] Liu Q, Kasuga M, Sakuma Y, Abe H, Miura S, Yamaguchi-Shinozaki K, Shinozaki K (1998). Two transcription factors, DREB1 and DREB2, with an EREBP/AP2 DNA binding domain separate two cellular signal transduction pathways in drought- and low-temperature-responsive gene expression, respectively, in Arabidopsis. Plant Cell.

[8] Gong Z, Xiong L, Shi H, Yang S, Herrera-Estrella LR, Xu G, Chao DY, Li J, Wang PY, Qin F (2020). Plant abiotic stress response and nutrient use efficiency. Sci China Life Sci.

[9] Lin Q, Xie Y, Guan W, Duan Y, Wang Z, Sun C (2019). Combined transcriptomic and proteomic analysis of cold stress induced sugar accumulation and heat shock proteins expression during postharvest potato tuber storage. Food Chem.

[10] Yue C, Cao HL, Wang L, Zhou YH, Huang YT, Hao XY, Wang YC, Wang B, Yang YJ, Wang XC (2015). Effects of cold acclimation on sugar metabolism and sugar-related gene expression in tea plant during the winter season. Plant Mol Biol.

[11] Huang S, Ding J, Deng D, Tang W, Sun H, Liu D, Zhang L, Niu X, Zhang X, Meng M (2013). Draft genome of the kiwifruit Actinidia chinensis. Nat Commun.

[12] Yue J, Liu J, Tang W, Wu YQ, Tang X, Li W, Yang Y, Wang L, Huang S, Fang C (2020). Kiwifruit genome database (KGD): a comprehensive resource for kiwifruit genomics. Horticulture Research.

[13] Mays AD, Schmidt M, Graham G, Tseng E, Baybayan P, Sebra R, Sanda M, Mazarati JB, Riegel A, Wellstein A (2019). Single-molecule real-time (SMRT) full-length RNA-sequencing reveals novel and distinct mRNA isoforms in human bone marrow cell subpopulations. Genes.

[14] Wang B, Kumar V, Olson A, Ware D (2019). Reviving the Transcriptome studies: an insight into the emergence of single-molecule Transcriptome sequencing. Front Genet.

[15] Eid J, Fehr A, Gray J, Luong K, Lyle J, Otto G, Peluso P, Rank D, Baybayan P, Bettman B (2009). Real-time DNA sequencing from single polymerase molecules. Science.

[16] Ma JE, Jiang HY, Li LM, Zhang XJ, Li HM, Li GY, Mo DY, Chen JP (2019). SMRT sequencing of the full-length transcriptome of the Sunda pangolin (Manis javanica). Gene.

[17] Gao J, Dai G, Zhou W, Liang H, Huang J, Qing D, Chen W, Wu H, Yang X, Li D (2019). Mapping and identifying a candidate gene Plr4, a recessive gene regulating purple leaf in Rice, by using bulked Segregant and Transcriptome analysis with next-generation sequencing. Int J Mol Sci.

[18] Du H, Zhu J, Su H, Huang M, Wang H, Ding S, Zhang B, Luo A, Wei S, Tian X (2017). Bulked Segregant RNA-seq reveals differential expression and SNPs of candidate genes associated with waterlogging tolerance in maize. Front Plant Sci.

[19] Catalá R, López-Cobollo R, Mar Castellano M, Angosto T, Alonso JM, Ecker JR, Salinas J (2014). The Arabidopsis 14-3-3 protein RARE COLD INDUCIBLE 1A links low-temperature response and ethylene biosynthesis to regulate freezing tolerance and cold acclimation. Plant Cell.

[20] Liu X, Bi B, Xu X, Li B, Tian S, Wang J, Zhang H, Wang G, Han Y, McElroy JS (2019). Rapid identification of a candidate nicosulfuron sensitivity gene (Nss) in maize (Zea mays L.) via combining bulked segregant analysis and RNA-seq. Theor Appl Genet.

[21] Tang H, Li J, Xing S, Du S, Wang Z, Sun L, Liu X (2015). RNA-seq and bulked Segregant analysis of a gene related to high growth in Ginkgo biloba half-siblings. Am J Plant Sci.

[22] Nishijima R, Yoshida K, Sakaguchi K, Yoshimura S-I, Sato K, Takumi S (2018). RNA sequencing-based bulked Segregant analysis facilitates efficient D-genome marker development for a specific chromosomal region of synthetic Hexaploid wheat. Int J Mol Sci.

[23] Zhen H, Gary P, Xunjia L, Abhinandan D, FK C, GB D, MM R, Fengqun Y. Fine Mapping of a Clubroot Resistance Gene in Chinese Cabbage Using SNP Markers Identified from Bulked Segregant RNA Sequencing. Front Plant Sci. 2017;8:1448. 10.3389/fpls.2017.01448 PMC5581393 28894454

[24] Huang Z, Peng G, Gossen BD, Yu F (2019). Fine mapping of a clubroot resistance gene from turnip using SNP markers identified from bulked segregant RNA-Seq. Mol Breed.

[25] Lim TK (2012). Actinidia arguta. Edible medicinal and non-medicinal plants: volume 1, fruits.

[26] Tang W, Zheng Y, Dong J, Yu J, Yue JY, Liu FF, Guo XH, Huang SX, Wisniewski M, Sun JQ, et al. Comprehensive Transcriptome profiling reveals long noncoding RNA expression and alternative splicing regulation during fruit development and ripening in kiwifruit (Actinidia chinensis). Front Plant Sci. 2016;7:335. 10.3389/fpls.2016.00335 PMC5007456 27594858

[27] Zhang AD, Wang WQ, Tong Y, Li MJ, Grierson D, Ferguson I, Chen KS, Yin XR (2018). Transcriptome analysis identifies a zinc finger protein regulating starch degradation in kiwifruit. Plant Physiol.

[28] Li W, Liu Y, Zeng S, Xiao G, Wang G, Wang Y, Peng M, Huang H (2015). Gene expression profiling of development and anthocyanin accumulation in kiwifruit (Actinidia chinensis) based on Transcriptome sequencing. PLoS One.

[29] Zhang JY, Huang SN, Mo ZH, Xuan JP, Jia XD, Wang G, Guo ZR: De novo transcriptome sequencing and comparative analysis of differentially expressed genes in kiwifruit under waterlogging stress. Mol Breed 2015, **35**(11).

[30] Wang T, Wang G, Jia ZH, Pan DL, Zhang JY, Guo ZR: Transcriptome Analysis of Kiwifruit in Response to Pseudomonas syringae pv. actinidiae Infection. Int J Mol Sci 2018, **19**(2). 10.3390/ijms19020373 PMC5855595 29373527

[31] Huang G, Gong S, Xu W, Li P, Zhang D, Qin L, Li W, Li X (2011). GhHyPRP4, a cotton gene encoding putative hybrid proline-rich protein, is preferentially expressed in leaves and involved in plant response to cold stress. Acta Biochim Biophys Sin.

[32] Goodwin W, Pallas JA, Jenkins GI (1996). Transcripts of a gene encoding a putative cell wall-plasma membrane linker protein are specifically cold-induced in Brassica napus. Plant Mol Biol.

[33] Zhang Y, Schläppi M (2007). Cold responsive EARLI1 type HyPRPs improve freezing survival of yeast cells and form higher order complexes in plants. Planta.

[34] Saikia B, Singh S, Debbarma J, Velmurugan N, Dekaboruah H, Arunkumar KP, Chikkaputtaiah C (2020). Multigene CRISPR/Cas9 genome editing of hybrid proline rich proteins (HyPRPs) for sustainable multi-stress tolerance in crops: the review of a promising approach. Physiol Mol Biol Plants.

[35] Peng T, Jia M-M, Liu J-H: RNAi-based functional elucidation of PtrPRP, a gene encoding a hybrid proline rich protein, in cold tolerance of *Poncirus trifoliata*. Front Plant Sci 2015, **6**(808). 10.3389/fpls.2015.00808 PMC4587090 26483822

[36] Londo JP, Kovaleski AP, Lillis JA (2018). Divergence in the transcriptional landscape between low temperature and freeze shock in cultivated grapevine (Vitis vinifera). Horticulture Research.

[37] Sitnicka D, Orzechowski S (2014). Cold-induced starch degradation in potato leaves — intercultivar differences in the gene expression and activity of key enzymes. Biol Plant.

[38] Monroe JD, Storm AR (2018). Review: the Arabidopsis β-amylase (BAM) gene family: diversity of form and function. Plant Sci.

[39] Thalmann M, Santelia D (2017). Starch as a determinant of plant fitness under abiotic stress. New Phytol.

[40] Peng T, Zhu X, Duan N, Liu J-H (2014). PtrBAM1, a β-amylase-coding gene of Poncirus trifoliata, is a CBF regulon member with function in cold tolerance by modulating soluble sugar levels. Plant Cell Environ.

[41] Kaplan F (2004). Guy CL: **beta-amylase induction and the protective role of maltose during temperature shock**. Plant Physiol.

[42] Nishizawa A, Yabuta Y, Shigeoka S (2008). Galactinol and raffinose constitute a novel function to protect plants from oxidative damage. Plant Physiol.

[43] Kong F, Deng Y, Zhou B, Wang G, Wang Y, Meng Q (2014). A chloroplast-targeted DnaJ protein contributes to maintenance of photosystem II under chilling stress. J Exp Bot.

[44] Yang T, Huang X-S (2018). Deep sequencing-based characterization of transcriptome of Pyrus ussuriensis in response to cold stress. Gene.

[45] Shi Y, Tian S, Hou L, Huang X, Zhang X, Guo H, Yang S: Ethylene signaling negatively regulates freezing tolerance by repressing expression of the *CBF * and type-A*ARR* genes in *Arabidopsis*. Plant Cell. 2012;24(6):2578. 10.1105/tpc.112.098640 PMC3406918 22706288

[46] Jiang B, Shi Y, Zhang X, Xin X, Qi L, Guo H, Li J, Yang S. PIF3 is a negative regulator of the *CBF* pathway and freezing tolerance in *Arabidopsis*. Proc Natl Acad Sci. 2017;114(32):E6695. 10.1073/pnas.1706226114 PMC5559041 28739888

[47] Liu Z, Jia Y, Ding Y, Shi Y, Li Z, Guo Y, Gong Z, Yang S (2017). Plasma membrane CRPK1-mediated phosphorylation of 14-3-3 proteins induces their nuclear import to fine-tune CBF signaling during cold response. Mol Cell.

[48] Peng T, Zhu XF, Fan QJ, Sun P, Liu JH (2011). Identification and characterization of low temperature stress responsive genes in Poncirus trifoliata by suppression subtractive hybridization. Gene.

[49] Janz D, Behnke K, Schnitzler J-P, Kanawati B, Schmitt-Kopplin P, Polle A (2010). Pathway analysis of the transcriptome and metabolome of salt sensitive and tolerant poplar species reveals evolutionary adaption of stress tolerance mechanisms. BMC Plant Biol.

[50] Liu S, Jiang Y (2010). Identification of differentially expressed genes under drought stress in perennial ryegrass. Physiol Plant.

[51] Sun S, Lin M, Qi X, Zhao J, Meng X, Fang J (2019). Determination of semi-lethal temperature of kiwifruit by elextrolyte leakage method. Northern horticulture.

[52] Zhang J-Y, Huang S-N, Mo Z-H, Xuan J-P, Jia X-D, Wang G, Guo Z-R (2015). De novo transcriptome sequencing and comparative analysis of differentially expressed genes in kiwifruit under waterlogging stress. Mol Breed.

[53] Apweiler R, Bairoch A, Wu CH, Barker WC, Boeckmann B, Ferro S, Gasteiger E, Huang H, Lopez R, Magrane M (2004). UniProt: the universal protein knowledgebase. Nucleic Acids Res.

[54] Ashburner M, Ball CA, Blake JA, Botstein D, Butler H, Cherry JM, Davis AP, Dolinski K, Dwight SS, Eppig JT (2000). Gene ontology: tool for the unification of biology. The Gene Ontology Consortium Nat Genet.

[55] Koonin EV, Fedorova ND, Jackson JD, Jacobs AR, Krylov DM, Makarova KS, Mazumder R, Mekhedov SL, Nikolskaya AN, Rao BS (2004). A comprehensive evolutionary classification of proteins encoded in complete eukaryotic genomes. Genome Biol.

[56] Finn RD, Bateman A, Clements J, Coggill P, Eberhardt RY, Eddy SR, Heger A, Hetherington K, Holm L, Mistry J (2014). Pfam: the protein families database. Nucleic Acids Res.

[57] Kanehisa M, Goto S, Kawashima S, Okuno Y, Hattori M (2004). The KEGG resource for deciphering the genome. Nucleic Acids Res.

[58] Deng YY, Li JQ, Wu SF, Zhu Y, Chen YW, He FC (2006). Integrated nr database in protein annotation system and its localization. Comput Eng.

[59] Langmead B, Trapnell C, Pop M, Salzberg SL (2009). Ultrafast and memory-efficient alignment of short DNA sequences to the human genome. Genome Biol.

[60] Li B, Dewey CN (2011). RSEM: accurate transcript quantification from RNA-Seq data with or without a reference genome. BMC Bioinformatics.

[61] Trapnell C, Williams BA, Pertea G, Mortazavi A, Kwan G, van Baren MJ, Salzberg SL, Wold BJ, Pachter L (2010). Transcript assembly and quantification by RNA-Seq reveals unannotated transcripts and isoform switching during cell differentiation. Nat Biotechnol.

[62] Wang L, Feng Z, Wang X, Wang X, Zhang X (2010). DEGseq: an R package for identifying differentially expressed genes from RNA-seq data. Bioinformatics.

[63] Dillies MA, Rau A, Aubert J, Hennequet-Antier C, Jeanmougin M, Servant N, Keime C, Marot G, Castel D, Estelle J (2013). A comprehensive evaluation of normalization methods for Illumina high-throughput RNA sequencing data analysis. Brief Bioinform.

[64] Young MD, Wakefield MJ, Smyth GK, Oshlack A (2010). Gene ontology analysis for RNA-seq: accounting for selection bias. Genome Biol.

[65] Mao X, Tao C, Olyarchuk JG, Wei L (2005). Automated genome annotation and pathway identification using the KEGG Orthology (KO) as a controlled vocabulary. Bioinformatics.

[66] Kanehisa M, Araki M, Goto S, Hattori M, Hirakawa M, Itoh M, Katayama T, Kawashima S, Okuda S, Tokimatsu T (2008). KEGG for linking genomes to life and the environment. Nucleic Acids Res.

