# Supplementary figures and images for: BSR-Seq analysis provides insights into the cold stress response of Actinidia arguta F1 populations
Source: BMC Genomics. 2021 Jan 22;22:72. doi: 10.1186/s12864-021-07369-9 (PMC7821520; doi:10.1186/s12864-021-07369-9)

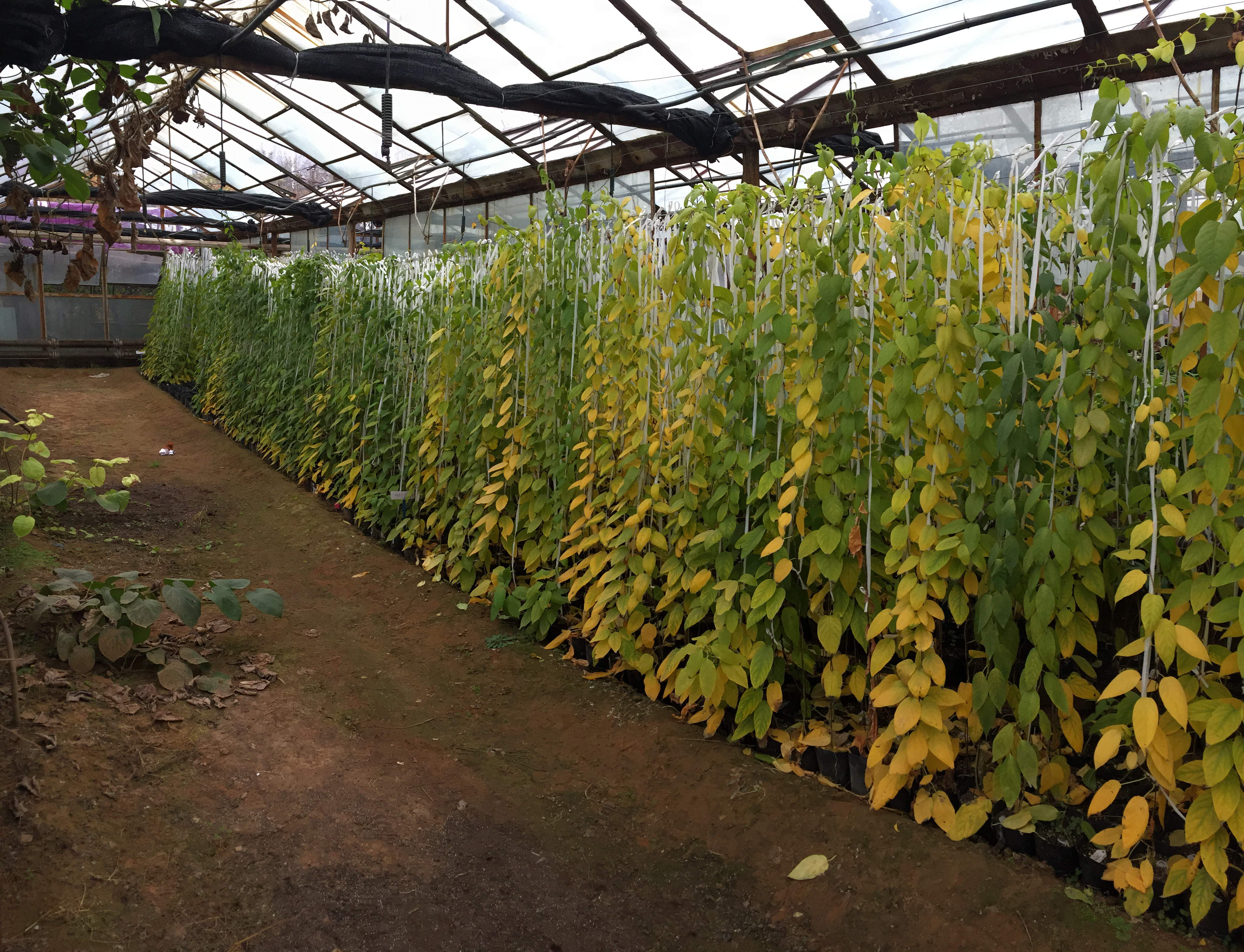

Supplement: Supplementary file 6 — Additional file 6: Fig. S1. The hybrid of ‘Ruby-3’ × ‘Kuilv’ male. [file 12864_2021_7369_MOESM6_ESM.tif]

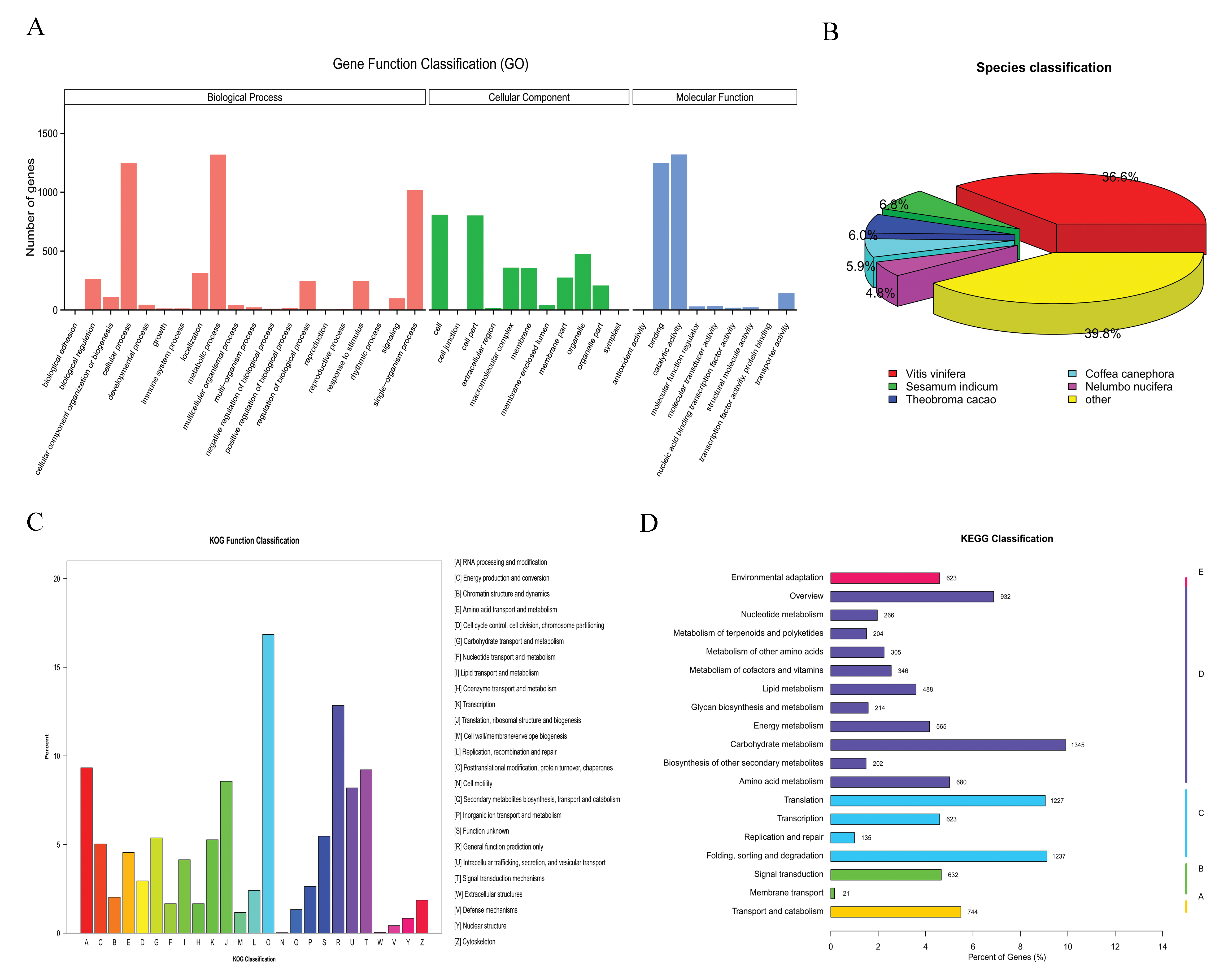

Supplement: Supplementary file 7 — Additional file 7: Fig. S2. Gene functional annotation of unigenes based on PacBio transcriptome data corrected by HiSeq 2000. A: GO classification, B: NR classification, C: KOG function classification, D: KEGG classification. [file 12864_2021_7369_MOESM7_ESM.tif]
